# Single-cell transcriptomic analysis reveals tumor cell heterogeneity and immune microenvironment features of pituitary neuroendocrine tumors

**DOI:** 10.1186/s13073-023-01267-3

**Published:** 2024-01-02

**Authors:** Nan Yan, Weiyan Xie, Dongfang Wang, Qiuyue Fang, Jing Guo, Yiyuan Chen, Xinqi Li, Lei Gong, Jialin Wang, Wenbo Guo, Xuegong Zhang, Yazhuo Zhang, Jin Gu, Chuzhong Li

**Affiliations:** 1 MOE Key Laboratory of Bioinformatics, Department of Automation, BNRIST Bioinformatics Division, Tsinghua University, Beijing, 100084 China; 2 Beijing Neurosurgical Institute, Capital Medical University, Beijing, 100070 China; 3 Biomedical Pioneering Innovative Center, Peking University, Beijing, 100871 China; 4 Department of Neurosurgery, Beijing Tiantan Hospital Affiliated to Capital Medical University, Beijing, 100070 China

**Keywords:** Pituitary neuroendocrine tumor, Tumor heterogeneity, Tumor microenvironment, Single-cell RNA sequencing

## Abstract

**Background:**

Pituitary neuroendocrine tumors (PitNETs) are one of the most common types of intracranial tumors. Currently, the cellular characteristics of normal pituitary and various other types of PitNETs are still not completely understood.

**Methods:**

We performed single-cell RNA sequencing (scRNA-seq) on 4 normal samples and 24 PitNET samples for comprehensive bioinformatics analysis. Findings regarding the function of *PBK* in the aggressive tumor cells were validated by siRNA knockdown, overexpression, and transwell experiments.

**Results:**

We first constructed a reference cell atlas of the human pituitary. Subsequent scRNA-seq analysis of PitNET samples, representing major tumor subtypes, shed light on the intrinsic cellular heterogeneities of the tumor cells and tumor microenvironment (TME). We found that the expression of hormone-encoding genes defined the major variations of the PIT1-lineage tumor cell transcriptomic heterogeneities. A sub-population of TPIT-lineage tumor cells highly expressing *GZMK* suggested a novel subtype of corticotroph tumors. In immune cells, we found two clusters of tumor-associated macrophages, which were both highly enriched in PitNETs but with distinct functional characteristics. In PitNETs, the stress response pathway was significantly activated in T cells. While a majority of these tumors are benign, our study unveils a common existence of aggressive tumor cells in the studied samples, which highly express a set of malignant signature genes. The following functional experiments confirmed the oncogenic role of selected up-regulated genes. The over-expression of *PBK* could promote both tumor cell proliferation and migration, and it was also significantly associated with poor prognosis in PitNET patients.

**Conclusions:**

Our data and analysis manifested the basic cell types in the normal pituitary and inherent heterogeneity of PitNETs, identified several features of the tumor immune microenvironments, and found a novel epithelial cell sub-population with aggressive signatures across all the studied cases.

**Supplementary Information:**

The online version contains supplementary material available at 10.1186/s13073-023-01267-3.

## Background

Pituitary is a functionally critical endocrine gland in humans that produces hormones to regulate multiple essential physiological processes. The anterior pituitary consists of five main cell types that secret specific hormones [1, 2]: somatotrophs (growth hormone, GH), lactotrophs (prolactin, PRL), thyrotrophs (thyroid-stimulating hormone, TSH), gonadotrophs (follicle-stimulating hormone, FSH, and luteinizing hormone, LH), and corticotrophs (adrenocorticotropic hormone, ACTH). Pituitary neuroendocrine tumors (PitNETs) arise from one or more hormone-producing cell types and are classified according to the expression of cell-specific transcription factors [3]. Somatotroph, lactotroph, and thyrotroph tumors are of PIT1 (known as *POU1F1*) lineage, corticotroph tumors are of TPIT (known as *TBX19*) lineage, and gonadotroph tumors are of SF1 (known as *NR5A1*) lineage [4–6]. Plurihormonal tumor express multiple transcription factors, while null cell tumor lacks any gene listed above [7, 8]. Most PitNETs are benign, but they can cause hormonal excess or local mass compression with adverse clinical outcomes [9–11]. The pathogenesis of many pituitary tumors remains poorly elucidated and no single molecular marker is sufficient to predict their behavior.

Single-cell RNA sequencing (scRNA-seq) is a powerful bio-technology for characterizing cellular diversity [12, 13], revealing complexity among multiple cell types [14] and deciphering the heterogeneity of various tumors [15, 16]. It has been applied to unravel the relationship between hormone specificity and cell plasticity in mouse pituitary [17] and to identify cell developmental trajectories in human fetal pituitaries [18]. A previous study of PitNETs has identified novel tumor-related genes at the single-cell resolution [19]. And another recent study has used scRNA-seq to compare PitNET subtypes with normal anterior pituitary cells and found that the tumor differentiation status was associated with long-term recurrence [20]. However, the tumor cell heterogeneity of different lineages and the tumor microenvironment still need further investigation.

In this study, we conducted scRNA-seq on 4 normal pituitary tissues and on major types of PitNETs from 24 patients to build a comprehensive transcriptional landscape of human normal pituitary as well as PitNETs. Our data and analysis provide a deep multifaceted understanding of cellular heterogeneity and tumor microenvironment features of PitNETs, which underscores the inherent complexity of PitNETs.

## Methods

### Patients and clinical samples

In this study, we collected fresh tissues of 4 normal pituitary samples and 24 PitNET cases from 26 patients who had undergone pituitary surgery at Beijing Tiantan Hospital in 2018 (Additional file 1: Figure S1, Additional file 2: Table S1). The 4 normal pituitary samples were taken from the endonasal endoscopy surgeries requiring partial pituitary resection and transposition. All diagnoses of PitNETs were confirmed by a multidisciplinary group consisting of neurosurgeons, neuroradiologists, and neuropathologists. The invasive PitNET criteria were based on three aspects: (1) MRI manifestations: Knosp classification grades 3 and 4, Hardy–Wilson classification grades 3 and 4 or stage D and E; (2) intraoperative findings: tumor invasion into the dura mater, cavernous sinus, bone, or subarachnoid space; (3) pathological confirmation of tumor invasion into surrounding tissues. The invasive sample must meet the MRI criteria and intraoperative findings or pathological confirmation.

All procedures performed with the use of samples obtained from patients were approved by IRB of Beijing Tiantan Hospital, Capital Medical University (KY 2018–053-02). All the patients signed informed consent.

### Single-cell preparations

Fresh pituitary adenoma tissues were minced with the Iris scissor into small pieces and digested for 30 min at 37 °C, 800 rpm, with a digestion solution containing PBS and collagenase II and IV (1.5 mg/ml, Gibco). The cell suspension was further filtered through 45-µm nylon mesh to remove cell aggregates and re-suspended in L15 with 10% FBS. Then, a second enzymatic digestion with accutase to dissociate the remaining cell clusters into single cells was performed. Finally, add L15 medium enriched with 10% fetal bovine serum (FBS).

### Single-cell RNA-seq

The chromium single-cell expression solution (10 × Genomics) was used to generate single-cell transcriptomes of digested pituitary adenomas. The single-cell suspensions were loaded onto the Chromium Controller (using Chromium i7 Multiplex Kit, Chromium Single Cell 3′Library and Gel Bead Kit v2 and Chromium Single Cell A Chip Kit) for estimated 5000 ~ 10,000 captured cells per library. The libraries were sequenced by HiSeq4000 (Illumina) (150 bp paired-end sequencing).

### Preprocessing of scRNA-seq data

Raw gene expression (UMI counts; UMIs, unique molecular identifiers) matrices for each sample were obtained by CellRanger (6.0). Given that both P6T and P8T had two scRNA-seq samples, we selected one with higher sequencing quality from each patient (P6T1, P8T2) for the subsequent analysis. The following steps were processed based on the R (4.2.1) package “Seurat” (4.1.1) [21]. We firstly filtered out low-quality cells for each sample. Four filters were used: cells with very low or high number of UMIs, cells with very low or high number of detected genes, cells with high percent of mitochondrial genes, and cells with high percent of dissociation-associated genes. In addition, we removed those genes that were detected in less than 3 cells in any sample. After these, the expression of each gene was normalized by dividing the sum of remaining UMI counts in the cell and then multiplying 10,000 to obtain the TPM-like values. To make the expression of all genes comparable, the TPM-like values were further logarithm transformed after adding a pseudo-count 1. We then combined cells from all samples as a single expression matrix, and used the function “FindVariableGenes” with “selection.method = ’vst” from Seurat, selecting 2000 highly variable genes for the downstream analysis (Additional file 2: Table S2). The expression matrix of highly variable genes was scaled after regressing out the total count of UMIs and the percent of mitochondrial genes. To further reduce noise and dimensions, principal component (PC) analysis was performed on the scaled data matrix of the selected genes with the first 30 PCs remained. Harmony [22] was then adopted to remove the batch effects between different samples (Additional file 1: Figure S2a, S3a). Clustering was performed by the “FindClusters” function, and UMAP was used for visualization. We systematically performed two rounds of clustering and the corresponding visualization, with the first round discerning the major cell types, including PIT1-positive pituitary cells, PIT1-negative pituitary cells, *PTTG1*-positive epithelial cells, other epithelial cells, stromal cells, and immune cells. The second round of clustering then further identified the fine-grained cell types for each major one. The diffusion map and RNA velocity analysis were performed based on the Python package “scanpy” [23] and “scvelo” [24] with default settings.

### Identification of differentially expressed genes and biomarkers

Differentially expressed genes of each cluster were identified by Wilcoxon rank-sum test implemented in Seurat. We first applied the function “FindAllMarkers” to identify DEGs for each cluster compared with all cells from the rest clusters and then filtered genes with high Logarithmic fold change (> 0.25) and low adjusted *P* value (< 0.01) (Additional file 2: Table S3). Moreover, we also checked specific known marker gene expression to validate the characteristics of each cluster. The top differentially expressed genes and the biomarkers of each cluster were selected for visualization in heatmaps or bubble plots.

### Calculation of dendrogram distance

We firstly selected the cells of clusters N01–N05, filtered the expression of TF genes, and reperformed scaling and PCA reduction on these cells. We next selected the first 20 PCs, calculated the Euclidean distance between the average of each cluster, and constructed the dendrogram based on hierarchical clustering.

### Comparison analysis for human versus mouse and rat

We downloaded the dataset GSE120410 [25, 26] and GSE132224 [27, 28] as reference for mouse and rat, respectively. We converted the gene names of mouse and rat into corresponding human gene names and filtered these three datasets (including our human pituitary samples) by their common genes (15,406 genes) for subsequent analysis. For the mouse and rat datasets, we filtered cells with at least 200 genes and at most 50% of mitochondrial genes. Similar preprocessing procedures were applied on the filtered human, mouse, and rat datasets to obtain the integrated atlas for comparison (Additional file 1: Figure S2c, d). Inspired by scmap [29], we represented each cluster in mouse or rat by its centroid (mean gene expression) and calculated the Pearson correlation coefficients between the gene expression of every single-cell in human normal pituitary samples and the centroid of the corresponding cell type in mouse or rat as similarity. The stemness, epithelial, and mesenchymal gene signatures were calculated as the average expression based on the marker gene score definitions presented in the previous study [18]. To make the single-cells comparable across different samples and species, we considered every cell to be composed of three states according to its corresponding signatures. Next, we used R package ggtern (v3.4.2) to visualize the relative proportional relationship as cell state scores in one ternary plot.

### Pseudo-bulk construction and similarity calculation

The pseudo-bulk of each sample was constructed by summing up the log-normalized expression of all cells in one sample. We performed scaling, PCA reduction, and UMAP for visualization. The first 20 PCs were selected to calculate the pairwise cosine similarity of all pseudo-bulk samples.

### Signature calculation for M1 and M2 phenotypes of macrophages and the functions of T cells

The gene sets for calculating M1 (classically activated) and M2 (alternatively activated) phenotypes of two groups of tumor-associated macrophages (TAMs) were from a previous article [30]. We calculated the average of log-normalized expression as the signature of cluster I09 and cluster I10 for comparison. The gene sets for calculating the functions of T cells were from another study [31]. We used the function “AddModuleScore” in Seurat to calculate the signature scores of T cells in normal and tumor samples.

### Pathway enrichment analysis

We employed over representation analysis (ORA) and gene set enrichment analysis (GSEA) [32] to identify gene sets that have significant differences between selected clusters. In detail, we first converted captured gene names by a gene symbol to gene entrezID conversion function “bitr” using “org.Hs.eg.db” database, and hence those genes could not be converted were filtered. In ORA, we ranked differentially expressed genes according to adjusted *P* value and used top 50 genes of the cluster as input. In GSEA, we calculated log2FC for all filtered genes using Seurat function “FoldChange” based on the cluster annotation and took them as input. The ORA and GSEA results were given by the function “enricher” and “GSEA” from an R package “clusterProfiler” [33], respectively. The gene sets for testing included KEGG [34] pathways, Gene Ontology [35, 36] (Biological Processes), and Hallmark [37] gene sets, collected in Molecular Signatures Database (MSigDB). In order to ensure the reliability of enrichment, only the pathway that were identified as significantly enriched via both ORA and GSEA methods in at least two gene sets from MSigDB were selected. We eventually used the GSEA curve plots tested on GO:BP and the core enrichment genes identified by ORA for visualization.

### Cell–cell communication analysis

We applied CellChat [38] package on all immune and stromal clusters for cellular interaction analysis. To compare the difference between tumor and normal pituitary samples, we divided every cluster into tumor and normal groups according to the source of cells. Then, we ran the pipeline of CellChat with default settings to analyze characteristics of cell–cell communication with respect to these two groups. Considering the relatively low number of some groups of clusters, we used the function “projectData” to project the inference results from gene expression to protein–protein interaction networks for higher reliability. After that, we merged the results from tumor and normal for comparative analyses.

### Cell cycle scoring

We used the function “CellCycleScoring” in Seurat package to calculate the cell cycle scores of all clusters in PitNETs. The gene set for G1, S, and G2M was from the previous study [39].

### Pan-cancer analysis

We calculated the logarithmic fold change based on the normalized median expression of several tumor types and the match normal tissue in TCGA and GTEx datasets, where the expression data was collected from GEPIA [40].

### Classification based on scRNA-seq data

It is hypothesized that the expression patterns of the aggressive cells from cluster T00 should be used to distinguish malignant pituitary carcinomas from the other benign tumors. Firstly, we selected important genes as input features by the following procedures: (1) “FindMarker” function provided 83 differentially expressed genes in cluster T00 (589 genes in total); (2) filter genes with average logarithmic fold change higher than 0.75 and adjusted *P* value lower than 0.05 (only 83 genes left, reflecting the highly importance of DEGs); (3) further reduce this list by filtering out those not detected in the bulk dataset (the number reduced to 32). These 32 genes were used in the following analysis.

To match the expression distribution of our scRNA-seq data and the bulk data in GSE22812 [41, 42] (13 pituitary adenomas with 3 carcinomas), we reconciled the two datasets on the same measure scale. For the scRNA-seq dataset, the logarithmic normalized expression matrix with 32 genes was scaled to *z*-scores. For bulk data, we applied the “impute.knn” function from an R package “impute” to impute the “NA” values and then “normalize.quantiles” from “preprocessCore” package to normalize bulk samples. Afterwards, each gene’s expression value was also transformed to *z*-scores by centering and scaling.

As our assumption that the bulk data and the scRNA-seq data should have been scaled to similar distributions with the above pre-processing steps, we therefore used the 32 genes to train a random forest classier based on the scRNA-seq data by the “randomForest” function from the “randomForest” R, where “ntree” parameter was set as 4000; “strata” parameter was set as true labels, and “sampsize” was set to aggressive vs non-aggressive as 1500 vs 500 for balanced training. The “predict” function, where “type” parameter was set as “prob,” eventually gave the probability of each bulk sample to be “non-aggressive” or “aggressive.”

### Progression-free survival curves analysis

We collected 50 clinical PitNET samples and classified them based on the PBK expressions by RNAscope. Kaplan–Meier survival curves were used to demonstrate the significant difference in prognosis. In our study, PFS analysis was defined as the time from surgery to the first diagnosis of regrowth. Patients who lost follow-up or study end dates were considered censored in comparative survival analyses. The progression-free survival outcome was estimated by Kaplan–Meier method, and the difference was analyzed by log-rank test. *P* value < 0.05 was considered statistically significant.

### Immunofluorescence staining

Utilizing the multiplex IHC technique, we performed immunofluorescence staining of human FFPE tissues with the PANO 7-plex IHC kit (catalog number 0004100100, Panovue, Beijing, China). The procedure involved the sequential application of various primary antibodies, including those specific for GZMK (HPA063181, Sigma-Aldrich, St. Louis, MO, USA) and TPIT (ZM-0318, ZSGB-Bio, Beijing, China). Following this, slides were incubated with a horseradish peroxidase-conjugated secondary antibody and subsequently underwent tyramide signal amplification (TSA). After each TSA step, the slides were subjected to microwave treatment. Once all human antigens were appropriately labeled, nuclei were stained using 4′-6′-diamidino-2-phenylindole (DAPI, provided by Sigma-Aldrich).

Hybridizations using the RNAscope method were performed according to the manufacturer’s protocol (Advanced Cell Diagnostics) using the RNAscope 2.5 HD Duplex Reagent Kit (322,430). Probes used were Hs-PBK (551,871).

### Electron microscopy

Immediately after tumor tissue excision, small blocks of tumor were fixed in a mixture of 2.5% glutaraldehyde and 2% paraformaldehyde for 2 h at 4 °C and then washed three times with 0.1 M phosphate buffer. They were then dehydrated in gradient concentrations of ethanol, finally embedding the samples in pure epoxy resin (Epon 812, Shanghai, China). Ultrathin sections from 60 to 100 nM for electron microscopy were double stained with uranyl acetate and lead citrate and examined under a Hitachi H-7650 transmission electron microscope (Tokyo, Japan).

### Cell culture

Rat pituitary cells (GH3) were originally obtained from the American Type Culture Collection (ATCC) and cultured at 37 °C in 35 mm dishes in a humidified atmosphere of 95% air and 5% CO_2_. The culture medium was Ham’s F12K medium with 2.5% fetal bovine serum (FBS) and 15% horse bovine serum. Cultures were fed every other day. The cell lines were also genotyped to rule out cross-contamination and their morphology was regularly examined.

### Transfection and RNA interference

Small interfering RNA (siRNA) transfections were performed using Lipofectamine 2000 (11,668,019, Thermo Fisher), according to the manufacturer’s protocol. siRNA synthesis was performed by Shanghai GenePharma and the siRNA sequences for human GBK and GGH are shown in Additional file 2: Table S4.

### Quantitative real-time PCR

Total RNA was extracted using RNeasy Mini Kit (76,104, Qiagen) and then reversed transcribed using High-Capacity cDNA Reverse Transcription Kit (0049472, Thermo Fisher) according to the manufacturer’s instructions. Subsequently, we performed qRT-PCR using Power SYBR™ Green PCR Master Mix (4,367,659, Thermo Fisher) in a total reaction volume of 10 μL. GAPDH was used as a reference gene. The levels of mRNAs were performed on an ABI 7500 System (Applied Biosystems). Primer pairs for quantitative real-time PCR are shown in Additional file 2: Table S4. Amplification was performed as follows: 95 °C for 10 min and 40 cycles at 95 °C for 15 s, 60 °C for 60 s. For the quantitative analysis, relative expression levels were calculated based on CT values (corrected for GAPDH expression) according to the equation: 2-△CT [△CT = CT (gene of interest) − CT (GAPDH)]. All qRT-PCR analyses were performed in triplicate. Expression levels after different siRNAs’ transfections were shown in Figure S10.

### CCK-8 assay cell growth viability

Cells after treated or untreated were seeded at a concentration of 4 × 10^3^ per well in the 96-well plate and cultured for 24 h, 48 h, 72 h, 96 h, and 120 h at 37 °C, 5% CO_2_. Each group were detected with Cell Counting Kit-8 (Beyotime, C0039), following the manufacturer’s instructions. Briefly, 10 µl CCK-8 was added into each well, and cells were incubated for additional 4 h. The absorbance at 450 nm was measured using a microplate reader.

### Apoptosis analysis

Cells were analyzed for apoptosis by an Annexin V-FITC/propidium iodide double-staining method described by kit manufacturer (Beyotime, C1062M). The cells 48 h after transfection of siRNAs or expression vector plasmids were collected and subjected to the analysis. About 5 × 10^5^ cells each group were collected by centrifugation and resuspended 500 μl of binding buffer. Five microliters of Annexin V-FITC and 5 μl of propidium iodide were added into each tube and then incubated at room temperature for 15 min in the dark. Stained cells were analyzed by flow cytometry in FITC and ECD channels.

### Plasmid

The Flag-tagged PBK construct was subcloned into the pHS-AVC vector. The pZDonor-CMV-PBK-3flag (Rat, NM_001079937) overexpression vector was purchased from SyngenTech (Beijing, China).

### Transfection and RNA interference

GH3 cells (1 × 10^6^ per well) were seeded in six-well plates. After 24 h of incubation in the humidified incubator, GH3 cells were transfected with small interfering RNA (siRNA) or plasmids using lipofectamine 3000 (Lipo3000, Thermo Fisher, USA), according to the product specification. The specific siRNAs against PBK were purchased from GenePharma (Suzhou, China). The sequences of PBK siRNAs are as follows:

- PBK-Rat-1, 5′ GGUAGUCUGUGCCUUGCUATT 3′
- 5′ UAGCAAGGCACAGACUACCTT 3′
- PBK-Rat-2, 5′ GCAUGGAGACAUAAAGUCUTT 3′
- 5′ AGACUUUAUGUCUCCAUGCTT 3′
- PBK-Rat-3, 5′ GGGUCAGUGUUUACCUAAUTT 3′
- 5′ AUUAGGUAAACACUGACCCTT 3′

### Western blotting

GH3 cells were lysed with RIPA buffer (NCM Biotech, China). The obtained protein concentration was determined using the BCA Protein Assay kit (Thermo Fisher, USA). Proteins were separated by SDS-PAGE and transferred to PVDF membranes (Millipore, USA). Membranes were blocked and incubated in diluted primary anti-PBK antibody (dil. 1:1000, Santa Cruz, USA) and anti-Flag (dil. 1:1000, Sigma-Aldrich, USA) at 4 °C overnight followed by secondary antibodies. Tubulin was used as the internal control, and the grey values were calculated with the ImageJ software.

### Transwell migration assay

GH3 cells (5 × 10^5^ per well) were seeded in the upper chambers in 24-well culture plates with 8-μm pores (Corning, USA). GH3 cells were allowed to migrate through the pores in the transwell membrane during incubation at 37 °C with 5% CO_2_ for 24 h. Then, cells on the lower surface of the membrane were fixed in 4% paraformaldehyde for 15 min, stained with crystal violet for 10 min, washed with PBS. The migrated GH3 cells were imaged under a microscope and counted using the ImageJ software.

## Results

### Transcriptomic analysis of the cell populations in human pituitary

**Fig. 1:**
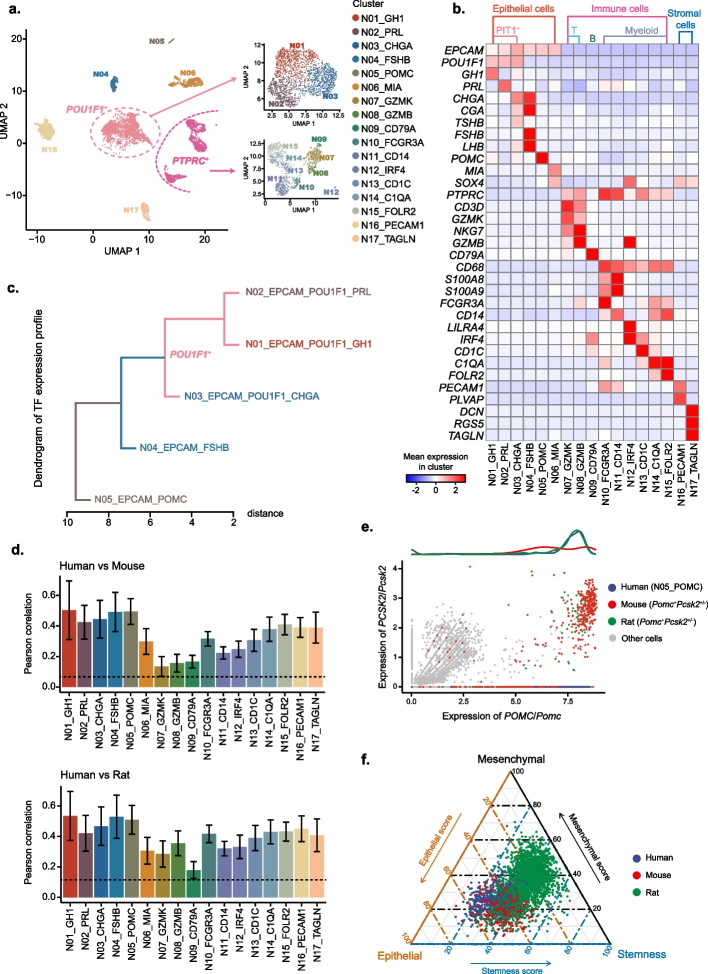
Single-cell landscape of normal human pituitary gland. **a** UMAP plot showing the annotated cell types from normal pituitary samples. Left: UMAP plot of all the clusters. Upper right: UMAP plot of only PIT1-positive epithelial cell clusters. Lower right: UMAP plot of only immune cell clusters. **b** Matrix plot showing mean expression of characteristic markers for each annotated cell types. **c** Dendrogram representing the similarity of TF expression profiles between different hormone-producing cell types. **d** Pearson correlation of human normal cell types with the corresponding cell types from other species. Up: mouse pituitary samples. Down: rat pituitary samples. The dashed lines represent the correlation between mean expression of all cells from human and those from mouse and rat, respectively. **e** Scatter plot showing few melanotrope (*POMC* + *PCSK2* +) cells detected in human samples. Both corticotrope and melanotrope exist in mouse and rat samples. **f** Ternary plot characterizing pituitary stem cells from different species by three kinds of signature scores

To construct a reference single-cell atlas of human pituitary, we integrated 5361 high-quality single cells from 4 normal pituitary samples, with an average of 11,172 UMIs and 2397 genes per cell. Unsupervised clustering was then performed, identifying 17 distinct cell populations (Fig. 1a), which in general could be categorized into 3 main types according to their representative marker genes (Fig. 1b, Additional file 1: Figure S2b): epithelial cells (N01-N06, including hormone-secreting cells and pituitary stem-like cells), immune cells (N07-N15, including T, B, myeloid cells), stromal cells (N16-N17, including fibroblasts and endothelial cells). As expected, epithelial cells accounted for the major compartment [43], and cell types of PIT1-lineage had the largest population. Further analyzing the regulatory similarity of hormone-secreting cells from different lineages, we found that the expression of the PIT1-positive cell clusters was more similar than those of PIT1-negative cells, where cells expressing *GH1* and *PRL* were the most similar (Fig. 1c). These similarities reflected the differentiation process of pituitary cells under the regulation of a set of transcription factors and was consistent with the fact that somatotrophs and lactotrophs were differentiated from the same precursor cells (mammosomatotrophs) [44].

Comparing our scRNA-seq data of human pituitary with mouse [25] and rat [27] pituitary, we corroborated that most cell types, especially epithelial and stromal cells, were highly conservative among these species, while immune cells had relatively low similarities (Fig. 1d, Additional file 1: Figure S2d). Notably, melanotrophs (N05), however, had different expression profiles (Fig. 1e), for both *Pomc* + /*Pcsk2* + and *Pomc* + /*Pcsk2* − cells were discovered in mice and rats while only *POMC* + /*PCSK2* − cells were found in human. Pituitary stem-like cells have previously been reported to possess a hybrid epithelial/mesenchymal state [18]. Based on the marker gene score definitions in this article [18], we calculated the epithelial, mesenchymal, and stemness scores of all cells in cluster N06 as well as the corresponding stem-like cells in mouse and rate to validate this transition situation. We observed that human and mouse had similar hybrid state, whereas rat had higher stemness and lower epithelial scores (Fig. 1f). This atlas comprehensively covers well-characterized pituitary cell types and provides a reference to analyze the altered cell populations and cellular states in PitNETs.

### Characterization of the cell populations in PitNETs

**Fig. 2:**
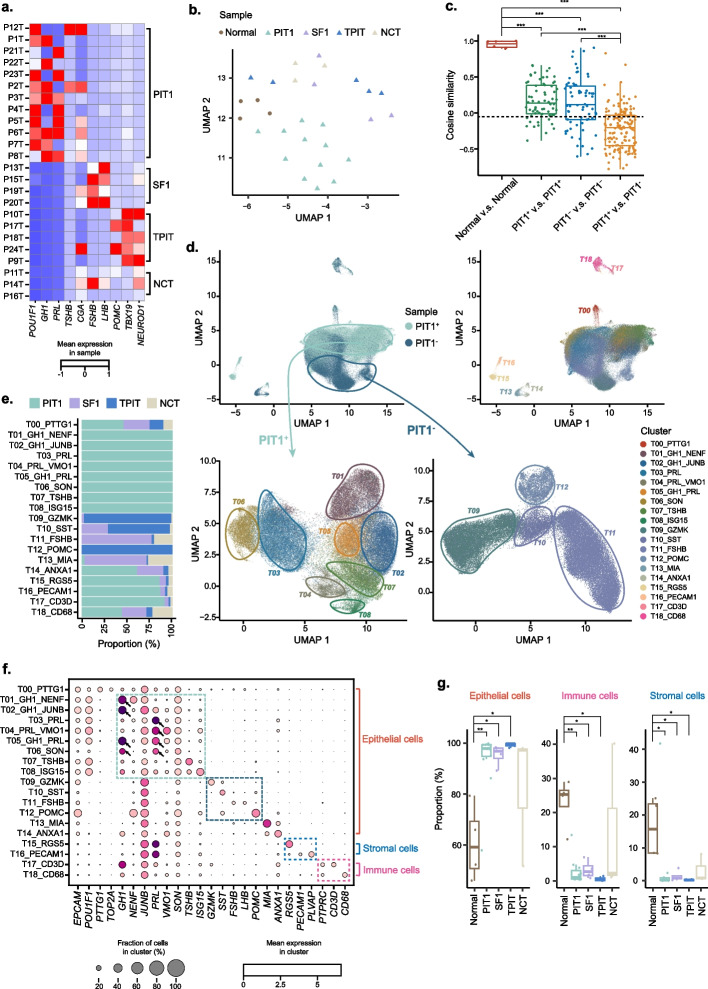
Single-cell landscape of PitNET samples. **a** Matrix plot showing mean expression of known markers for each tumor sample. Samples are arranged by the classification of PitNETs. **b** UMAP plot showing the distribution of pseudo-bulk expression from all samples. **c** Boxplot showing pairwise similarities between pseudo-bulk expression profiles from different kinds of samples. The dashed line represents the mean correlation among all PitNET samples. ***P < 0.001, Student’s *t* test. **d** UMAP plot characterizing clusters of all cells from tumor samples. Upper left: UMAP plot labeled by the sample source. Upper right: UMAP plot labeled by all clusters. Lower left: UMAP plot of only epithelial cells in PIT1-positive samples. Lower right: UMAP plot of only epithelial cells in PIT1-negative samples. **e** The proportion of cells from four types of PitNETs for each cluster. **f** Bubble heatmap showing the characteristic marker genes of each cluster. **g** Boxplot showing the proportion of major cell types across different kinds of sample. *P < 0.05, **P < 0.01, Wilcoxon rank sum test. PIT1, PIT1-lineage PitNETs; SF1, SF1-lineage PitNETs; TPIT, TPIT-lineage PitNETs; NCT, null cell tumor

We next collected 24 clinical samples from PitNET patients and obtained the scRNA-seq data of 126,483 cells in total after careful quality control. The samples contained four major types of PitNETs according to clinical diagnosis: PIT1-lineage PitNET, SF1-lineage PitNET, TPIT-lineage PitNET, and null cell tumor (NCT) (Fig. 2a, Additional file 1: Figure S1, Additional file 2: Table S1). We firstly constructed pseudo-bulk samples based on the corresponding scRNA-seq data for sample-wise analysis. Consistent with the known facts, *POU1F1* together with *GH1*, *PRL*, and/or *TSHB* were overexpressed in PIT1-lineage tumors; *FSHB* and/or *LHB* were overexpressed in SF1-lineage tumors; *TBX19* was overexpressed in TPIT-lineage tumors; and few transcription factors or hormone genes were captured in null cell tumors (Fig. 2a). The gene expression profiles in normal pituitary samples showed high similarity, yet the expression profiles of tumor samples differed greatly from each other, indicating strong inter-tumor heterogeneities (Fig. 2b, c and Additional file 1: Fig. S4). Nonetheless, we found that PIT1-lineage tumors were relatively similar, and the other non-PIT1-lineage tumors were also similar. However, there was not high degree of similarity between these two groups (Fig. 2c). To better represent the difference and commonality of PitNETs, therefore, we defined PIT1-lineage PitNETs as PIT1-positive pituitary tumors and the other non-PIT1-lineage PitNETs as PIT1-negative tumors for the following analysis.

By unsupervised clustering, the tumor cells and the major micro-environmental cell populations could be readily separated by a few typical marker genes. We observed that cell-types of immune cell and stromal cell were shared by both PIT1 positive and negative tumors, including fibroblasts (T15), endothelial cells (T16), and immune cells (T17, T18) (Fig. 2d, e). The tumor cells were largely distinguished according to hormone-encoded genes: the cells with high expression of only somatotropin (T01, T02), high expression of only prolactin (T03, T04), and high expression of both hormones (T05, T06) were found in PIT1-positive tumors, while the cells with high expression of gonadotropin (T11) and melanocortin (T12) were found in PIT1-negative tumors (Fig. 2e, f). We also discovered two interesting clusters of PIT1-negative tumors (T09, T10): the two clusters merely expressed hormone-encoding genes (*POMC*, *LHB*, *FSHB*) but highly expressed *GZMK* and *TBX19* in cluster T09 and *SST* in cluster T10. Compared to the previously established normal reference, the proportion of epithelial cells was significantly higher in PIT1, SF1, and TPIT lineage tumors, which suggested possible abnormal proliferation of tumor cells (Fig. 2g).

### Analysis of the PIT1-positive pituitary tumor cells

**Fig. 3:**
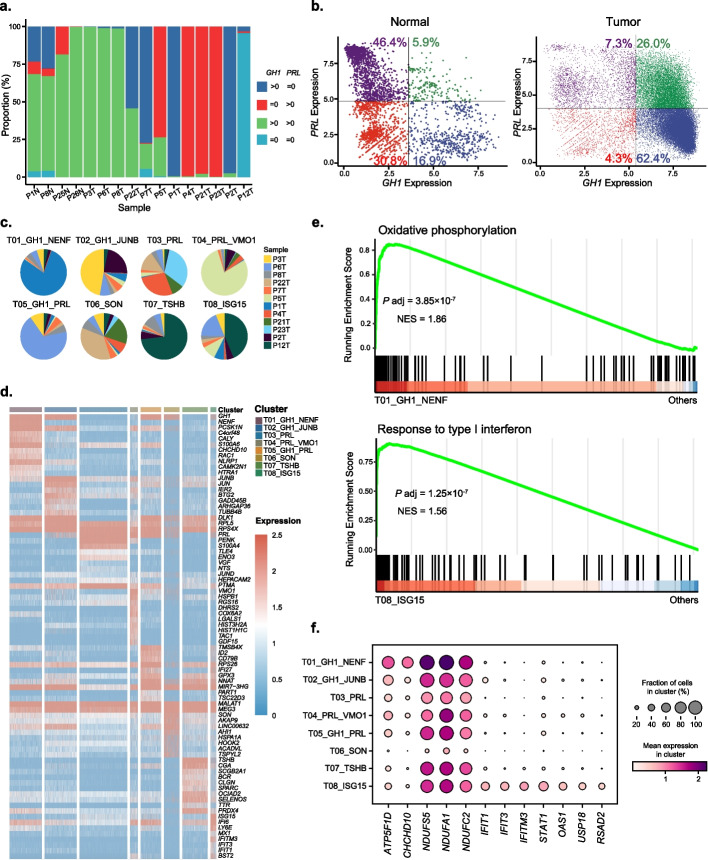
PIT1-positive tumor epithelial cells. **a** The proportion of cells with different *PRL* and *GH1* expression for each sample. **b** Scatter plots showing the expression relationship between *PRL* and *GH1* in normal and tumor cells. **c** The proportion of cells from PIT1-positive samples for each cluster. **d** Heatmap showing the expression of differentially expressed genes in each cluster. **e** GSEA plots showing different pathways enriched in clusters T01 and T08. **f** Bubble heatmap showing the corresponding pathway marker genes of each cluster

In this section, we focused on the tumor cells from the PIT1-positive pituitary tumors. Based on the expression of *GH1* and *PRL*, we observed that most cells in normal samples expressed both genes (Fig. 3a). Many tumor cases, however, only expressed one of these two hormone-encoding genes. Samples P1T and P2T, for example, mainly expressed *GH1* but few *PRL*, while samples P4T, P21T, and P23T strongly expressed *PRL* but few *GH1* (Fig. 3a). These results suggested that the epithelial cells in PIT1-positive tumors were altered to differentiate in only one direction in these tumors. We also found that the cells expressing both *GH1* and *PRL* had different expression patterns between the normal and tumor samples. In normal samples, the majority of these cells lowly expressed the two genes (low vs high as 30.8% vs 5.9%), whereas the cells in tumor samples tended to highly express both genes (low vs high as 4.3% vs 26.0%) (Fig. 3b). Transmission electron microscopy experiments confirmed the existence of both somatotropin and prolactin particles in the same tumor cells [45] (Additional file 1: Figure S5).

Our analysis revealed that PIT1-positive pituitary tumors exhibited strong inter-tumor heterogeneity similar to other malignant tumors, despite their non-aggressive nature and hormone-oriented classification rules. The majority of tumor cell clusters were predominantly composed of only one or two tumor samples (Fig. 3c). The differentially expressed markers among the clusters suggested that the heterogeneity mainly came from the hormone-encoding genes and their regulators (Fig. 3d). Except for these genes, we also found some differentially enriched functional signatures by gene set enrichment analysis (GSEA), such as “oxidative phosphorylation” for T01 (*ATP5F1D*, *CHCHD10*, *NDUFS5*) and “response to type I interferon” (*IFIT1*, *IFIT3*, *IFITM3*) for T08 (Fig. 3e, f).

### Analysis of the PIT1-negative pituitary tumor cells

**Fig. 4:**
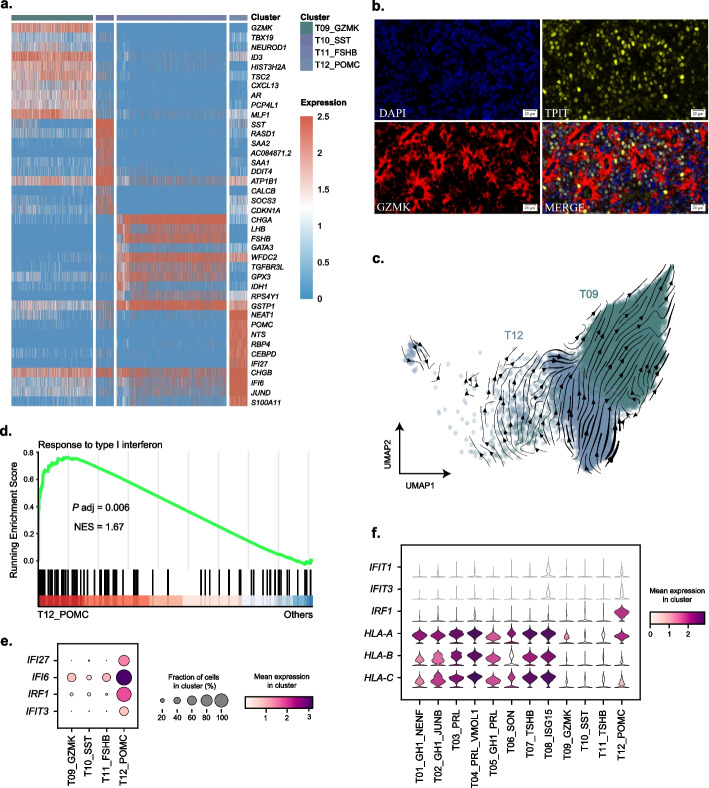
PIT1-negative tumor epithelial cells. **a** Heatmap showing the expression of differentially expressed genes in each cluster. **b** Multiplex immunohistochemical staining of GZMK (red) and TPIT (yellow) in corticotroph tumors. The thickness of the sections is 4 μm. Scale bar: 20 μm. **c** Inferred developmental trajectory of clusters T09 and T12 by RNA velocity. **d** GSEA plot showing the pathway enriched in cluster T12 over other clusters. **e** Bubble heatmap showing the pathway marker genes of each cluster. **f** Violin plots showing the expression of response to type I interferon pathway marker genes of each cluster

For the epithelial cells from non-PIT1-lineage PitNETs, we identified four major clusters (Fig. 4a). Two clusters highly expressed hormone-encoding genes: cluster T11 for *FSHB*/*LHB* and cluster T12 for *POMC*, while the other two clusters (T09, T10) only weakly expressed one hormone-encoding gene: *POMC* (Fig. 4a). It is known that *TBX19* (T-box transcription factor 19, TPIT) and *NEUROD1* (neuronal differentiation 1) are key transcription factor genes for the lineage specification of the *POMC* expressing cells [46–48]. Both genes were highly expressed in cluster T09 (Fig. 4a), indicating that this cluster was related to a subtype of corticotroph tumor. The multiplex immunofluorescence staining of *GZMK* in TPIT-lineage tumor cells also validate the existence of this cluster (Fig. 4b). Of note, the co-expression per cell quantified by immunofluorescence staining is pretty close to the co-expression rate calculated by scRNA-seq data (14.10% and 15.62%, respectively). Furthermore, two recent studies [19, 20] have also shown *GZMK* being one of the top DEGs in TPIT-lineage PitNETs. Taken together, these findings suggested a subpopulation with high expression of *GZMK* in corticotroph tumors.

Interestingly, cluster T12 exhibited high expression levels of *POMC* but low expression of the two transcription factors of corticotroph tumor, in contrast to cluster T09. Therefore, we utilized RNA velocity to examine the relationship between clusters T09 and T12, and the result showed that cluster T09 could be a non-functional degradation of cluster T12 (Fig. 4c). This revealed the possible mechanism of tumor formation of silent corticotroph tumor, a type of high risk PitNETs [49].

Cluster T10 barely expressed hormone-encoding genes, and was marked by *SST* (a growth hormone release-inhibiting factor) and *SAA2* (a potential biomarker for certain tumors). It suggested that this cluster could be a subtype of null cell tumor (Fig. 4a).

The GSEA analysis found a significant enrichment of “response to type I interferon” in cluster T12 over the other clusters (Fig. 4d), marked by the expression of *IFI27*, *IFI6*, *IRF1*, and *IFIT3* (Fig. 4e). Furthermore, we selected specific genes within this pathway to compare differences between PIT1-positive and PIT1-negative tumors. Our analysis revealed that HLA class I genes (*HLA-A*, *HLA-B*, *HLA-C*) were highly expressed in all clusters of PIT1-positive tumors and cluster T12 of PIT1-negative tumors (Fig. 4f). This might suggest more active endogenous antigen-presentation in PIT1 lineage PitNETs.

### Characterization of tumor-associated immune cells and stromal cells

**Fig. 5:**
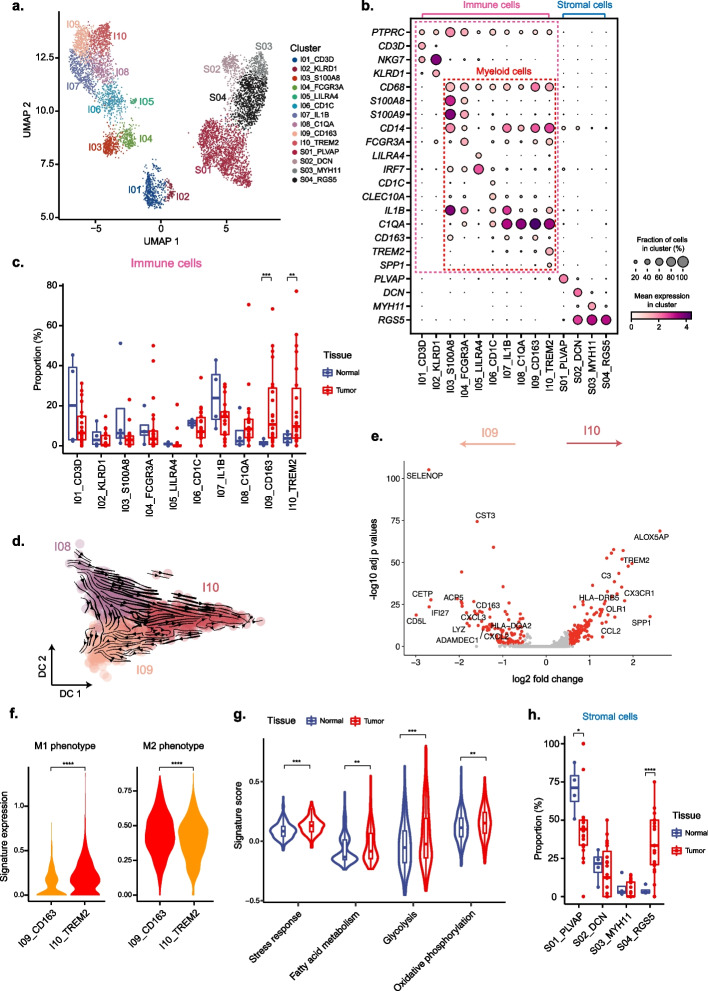
Characterization of tumor-infiltrating immune cells and stromal cells in normal and PitNET samples. **a** UMAP plot showing clusters of immune cells and stromal cells from all samples. **b** Bubble heatmap showing the characteristic marker genes of each cluster. **c** Boxplots showing the changes of immune cell clusters between normal and tumor samples. **d** Inferred developmental trajectory of tumor-enriched myeloid cell types by RNA velocity. **e** Volcano plot showing differentially expressed genes between two tumor-enriched macrophage clusters. **f** Boxplots showing comparison of M1 and M2 phenotype signature across indicated macrophage clusters. Cluster filled in red has higher mean expression. Student’s *t* test. **g** Comparison of selected T cell function signatures between normal and tumor samples. **h** Boxplots showing the changes of stromal cell clusters between normal and tumor samples. *P < 0.05, **P < 0.01, ***P < 0.001, ****P < 0.0001, Student’s *t* test

To investigate the microenvironment of PitNETs, we re-performed unsupervised clustering on 3335 immune cells and 3429 stromal cells from all normal and tumor samples. We identified 10 and 4 clusters for immune cells and stromal cells, respectively (Fig. 5a, b). Based on known markers, the immune cell clusters were annotated as T cells (I01), NK cells (I02), monocyte-like cells (I03 with CD14-positive, I04 with CD16-positive), dendritic cells (plasmacytoid dendritic cell like I05, conventional dendritic cell like I06), and macrophages (I07-I10). The stromal cells included endothelial cells (S01), fibroblasts (S02), smooth muscle cells (S03), and pericytes (S04).

Most immune cell clusters (I01-I08) were shared in both normal and tumor tissues, whereas 2 clusters of macrophages (I09, I10) were significantly enriched in the tumors, revealing the existence of tumor-associated macrophages (TAMs) in the PitNETs (Fig. 5c). Further comparisons across different types of PitNETs showed that T cells (I01) tended to be enriched in PIT1-lineage and TPIT-lineage tumors, while NK cells (I02) were more likely to be highly enriched only in PIT1-lineage PitNETs (Additional file 1: Figure S6). It was also notable to found that one group of TAMs (I10) differentially distributed across different types of PitNETs (Additional file 1: Figure S6).

Next, we analyzed the differentiation trajectory and molecular features of the tumor-enriched clusters of macrophages (I08-I10). The RNA velocity result showed that cluster I08 had the transition tendency to cluster I09 and cluster I10 (Fig. 5d), implying that these TAMs could be two differentiation endpoints of macrophages in PitNETs. Highly expressed genes in cluster I09 included *HLA-DQA2*, an HLA class II gene for exogenous antigen presentation, and *CXCL3*, a chemokine family member gene for the migration and adhesion of monocytes. By contrast, highly expressed genes in cluster I10 included multiple known pro-tumor markers in macrophages, such as *TREM2*, *CX3CR1*, and *SPP1* (Fig. 5e). Moreover, cluster I09 had significantly higher M2 phenotype signature, while cluster I10 had higher M1 phenotype signature (Fig. 5f).

We also characterized the functions of T cells based on the curated gene signatures from the recent study [31] and found significantly higher signature of stress response, glycolysis, fatty acid metabolism, and oxidative phosphorylation in PitNETs (Fig. 5g, Additional file 1: Figure S7). This observation implied that PitNETs might develop with resistance to immunotherapy [31]. In addition, we briefly analyzed the difference of cell–cell communications between PitNETs and normal samples. As a recent work has reported decreased cell–cell communication from “benign” to “malignant” PIT1-positive pituitary tumor [43], it is interesting to further discover that both the number and strength of cellular interactions are greatly lower in the PitNETs against normal tissues (Additional file 1: Figure S8a). Nevertheless, one group of TAMs (I09) still showed a more active communication with all immune and stromal subpopulations in PitNETs (Additional file 1: Figure S8b). In stromal cells, the pericytes accounted for a significantly larger proportion in tumor than the normal tissue, while the proportion of endothelial cells decreased in tumors (Fig. 5h). These changes implied that the PitNET cells could be shielded by increased pericytes coverage [50, 51]. Unlike other types of tumors, we found no significant difference in the proportion of fibroblasts between normal and tumor tissues (Fig. 5h).

### Analysis on the aggressive tumor cell sub-population

**Fig. 6:**
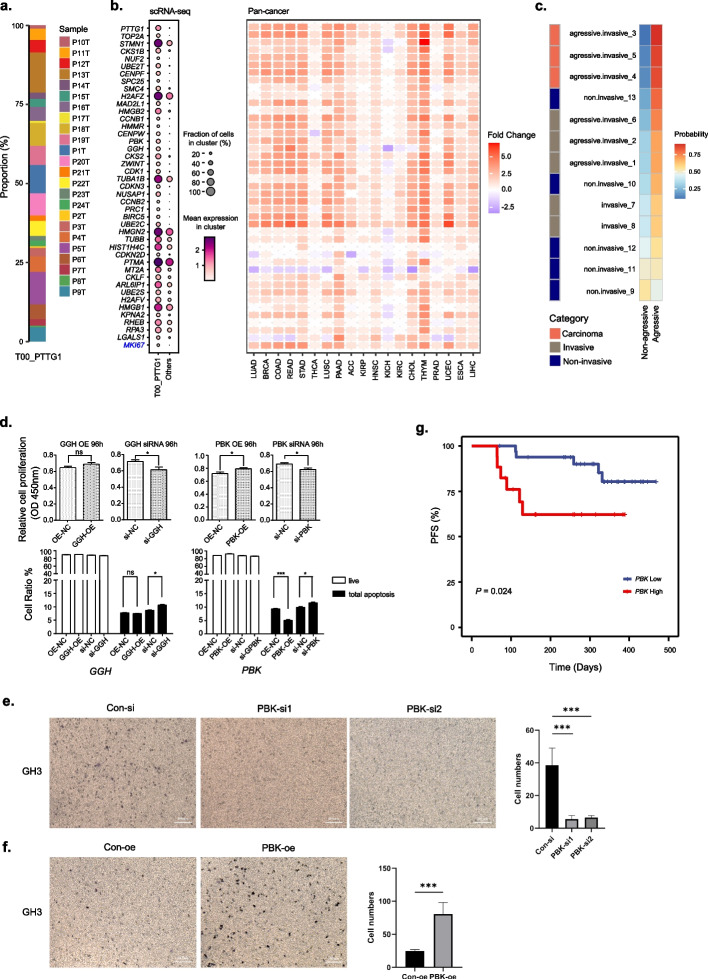
Aggressive tumor cell sub-populations. **a** The proportion of cells from each tumor sample for cluster T00. **b** Heatmap showing the highly expressed genes of cluster T00 in scRNA-seq data (left) and pan-cancer RNA-Seq data (right). *MKI67* in blue serves as a selected differential marker for comparison, though it does not rank at the top. The fold change stands for tumors against adjacent normal tissues in TCGA. **c** The predicted aggressive degrees of the bulk pituitary tumor dataset by the random forest model. The left color panel stands for the clinical diagnosis of the samples. **d** Functional analyses by over-expressing and siRNA knockdown of *GGH* and *PBK*. The top panel stands for cell proliferation and the bottom panel for apoptosis. *P < 0.05, ***P < 0.001, ns, not significant, Student’s *t* test or one-way ANOVA. **e**, **f** Transwell migration analyses showed that *PBK* knockdown/overexpression would inhibited/promoted cell migration in GH3 cells, respectively. Left panel: representative images of transwell migration assay (scale bar: 100 μm). Right panel: quantitative analysis in different groups. **P < 0.01, ***P < 0.001. Student’s *t* test or one-way ANOVA. **g** Progression-free survival curves of PitNET cases with low (RNAscope counting <  = 50) and high (RNAscope counting > 50) *PBK* expression. Log-rank test

Though most PitNETs are regarded as benign, the inter-tumor clustering analysis identify a population of 1947 “aggressive” tumor cells (T00) across all the tumor samples, with a median of 73.5 cells in 24 patients (Fig. 6a). Cluster T00 highly expressed several known aggressive tumor markers (such as *PTTG1*, *TOP2A*) in comparison with other tumor epithelial clusters (Fig. 6b) due to the higher proportion of cells in proliferation status (Additional file 1: Figure S9a, b). The abundance of cluster T00 in PitNET tissue was also found to be positively correlated to Ki-67 index examined in clinical (Additional file 1: Figure S9c). The great majority of these differentially expressed genes are consistently up-regulated in nearly all solid tumors according to the TCGA and GTEx pan-cancer dataset (Fig. 6b). To validate the malignancy of these cells, we additionally built a random forest-based machine learning model to learn the features to discriminate cluster T00 from other tumor cells. The trained model together with its corresponding learned gene features was then used to assess the aggressive degree of 13 pituitary tumor samples from an independent bulk gene expression dataset [41]. Three carcinoma samples in the bulk dataset were predicted as the most aggressive, and the other five invasive adenomas were also predicted to rank relatively higher than non-invasive samples in terms of the probability of being aggressive (Fig. 6c). Such consistency of our model prediction with the clinical diagnosis indicated that this cluster of tumor cells had the characteristic of malignancy.

To further elucidate the roles of up-regulated genes identified within this cluster, we focused on two specific genes, *PBK* and *GGH*. While these genes are established as biomarkers in a variety of tumor types [52, 53], their functional significance in PitNETs remains understudied. Through rigorous functional validations, we observed that both *GGH* and *PBK* significantly inhibited pituitary tumor cell apoptosis and enhanced cell proliferation (Fig. 6d, Additional file 1: Figure S10). Notably, further transwell experiments demonstrated that *PBK* also significantly increased the migration of GH3 pituitary tumor cells, implying the potential role in facilitating tumor cell invasiveness (Fig. 6e, f and Additional file 1: Figure S11). Moreover, we divided 50 clinical cases of PitNETs into two groups based on their *PBK* expressions. Patients with high *PBK* expression showed a prominent unfavorable prognosis than those with low *PBK* expression (Fig. 6g, Additional file 1: Figure S12). Taken together, these results provided further evidence for the malignancy nature of these commonly existing aggressive cells, and suggested potential prognostic value.

## Discussion

PitNETs are one of the most common types of intracranial tumors which require thorough investigations on the transcriptomic features at single-cell level. In a previous study, Cui et al. [19] have revealed the transcriptomic heterogeneity of PitNETs using scRNA-seq and found several tumor-related genes. Another study carried by Zhang et al. [20] has leveraged scRNA-seq data from normal pituitary and different types of PitNETs to characterize the cell sub-populations from a novel view of differentiation status. It also identified multiple differentiation-related markers and demonstrated their predictive value for the tumor recurrence. Our study also observed diverse tumor cell heterogeneity by constructing a single-cell-resolution transcriptomic atlas of human normal pituitary and major types of PitNETs. It is an interesting finding that *GZMK* is highly expressed in a subpopulation of corticotroph tumor cells, and similar results could also be observed in the previous studies [19, 20]. Trajectory inference implies new mechanism for the initialization of this tumor cellular subpopulation. The molecular characteristics of these corticotroph tumor cells require further investigations.

Apart from depicting tumor cell heterogeneity, our comprehensive analysis on immune cell subpopulations, function, and cellular interactions enables a more in-depth understanding of TME in PitNETs. We did not observe much immune infiltration in our clinical PitNET samples, and thereby no specific optimization of CD45 + cell sorting was performed at the beginning of our study, leading to a relatively limited number of immune cells captured. Nevertheless, major immune cell types are identified in the PitNET microenvironment. In contrast to the study by Lyu et al. [43], which has reported tumor infiltrating lymphocytes being the most abundant immune subtype in PIT1-positive pituitary tumors, our results show that myeloid cells account for the major proportion, including PIT1-lineage tumors.

Given that invasiveness is one of the most clinically important features, we have additionally performed detailed analyses about the invasive and non-invasive cases in our data. Only a slight difference is actually found in the composition of major cell types, T cell functions, and cellular interactions. Part of the reason is that the pathological and transcriptomic characteristics of invasive PitNETs vary greatly among individuals. This requires a larger sample size of single-cell sequencing data to explore the mechanisms of PitNET invasion, and we expect future research to address this question.

The majority of PitNETs are benign, but a few cases have an aggressive phenotype, with tumor tissue invading cavernous sinuses and parasellar structures with poor surgical results, and resistant to medical treatment or radiotherapy [54–56]. We identify a cluster of aggressive cells with potentially high proliferative capacity in all PitNET samples. These cells exhibit high expression level of multiple known pan-cancer proliferation markers. A random forest trained on these aggressive tumor cells can accurately distinguish pituitary carcinomas from adenomas, indicating that the cluster could contribute to an enhanced malignancy of PitNETs. Analyzing features of these cells may provide an opportunity to explore novel therapeutic strategies, provisionally by a few computational methods [57–59]. Given that only a few malignant markers have been specifically proposed in PitNETs so far, our study identifies a new gene *PBK* which is associated with cell proliferation, migration, and patient prognosis. Yet, other functional roles of *PBK*, such as cell invasion, still need more investigations. The finding of such aggressive tumor cells could open a way to study the cellular basis of malignant transformation of PitNETs.

In summary, our established single-cell atlas provides a systematical understanding of the inherent complexity of PitNETs and offers a refined perspective for molecular classification that complements traditional histopathological methods. Our study holds the potential to identify more signature genes which can serve as both candidate markers or novel therapeutic targets of different types of PitNETs. In the future, we plan to integrate such molecular findings with clinical outcomes to offer more translational insights for PitNETs.

## Conclusions

We firstly constructed the single-cell atlas of human normal pituitary, including epithelial cells, immune cells, and stromal cells as well as their subtypes. Since there are few datasets of normal pituitary in current human single-cell database, this complete single-cell atlas provides a precious reference resource for the community. Moreover, we obtained scRNA-seq data from 24 clinical samples of PitNETs, covering the major types of PitNETs, and performed comprehensive analyses to understand the cellular heterogeneity of the tumor cells and tumor microenvironment. The inter-tumor analysis showed great heterogeneity in gene signatures, hormone productions, and functional pathways within epithelial cells in PitNETs. For immune cells, we identified two clusters of tumor-associated macrophages with distinct functional characteristics. We also discovered significantly higher activation of the stress response pathway of T cells in PitNETs. While PitNETs are mostly benign in comparison with other solid tumors, it is important to unveil a common existence of aggressive tumor cells characterized by a set of malignant signature genes features in the studied samples. We conducted functional experiments to confirm the oncogenic role of selected up-regulated genes. The over-expression of *PBK* can promote both proliferation and migration capacity of PitNET cells, and it is also found to be associated with poor prognosis in PitNET patients. Our data together with the analyses could play a fundamental role in future research on human pituitary and provide comprehensive understanding about the complexity and inherent heterogeneity within PitNETs.

### Supplementary Information

**Additional file 1: Figure S1-S12.** All supplementary figures.

**Additional file 2:** **Table S1.** Sample information. **Table S2.** Highly variable genes used for clustering and visualizations. **Table S3.** Differentially expressed genes of each cluster in PitNETs. **Table S4.** Primers for qRT-PCR and sequences of siRNAs.

## Data Availability

The raw sequence data reported in this paper have been deposited in the Genome Sequence Archive [60] in National Genomics Data Center [61], China National Center for Bioinformation/Beijing Institute of Genomics, Chinese Academy of Sciences (GSA-Human: HRA003483) that are available at https://ngdc.cncb.ac.cn/gsa-human/browse/HRA003483 [62]. The processed annotation files are available at the URL: http://lifeome.net/supp/pituitary. The datasets analyzed during the study are available in the GEO repository: GSE120410 (https://www.ncbi.nlm.nih.gov/geo/query/acc.cgi?acc=GSE120410) [26] GSE132224 (https://www.ncbi.nlm.nih.gov/geo/query/acc.cgi?acc=GSE132224) [28] GSE22812 (https://www.ncbi.nlm.nih.gov/geo/query/acc.cgi?acc=GSE22812) [42]

## Abbreviations

ACTH: Adrenocorticotropic hormone
CCK-8: Cell Counting Kit-8
CT: Cycle threshold
DEGs: Differentially expressed genes
FBS: Fetal bovine serum
FSH: Follicle-stimulating hormone
GAPDH: Glyceraldehyde-3-phosphate dehydrogenase
GH: Growth hormone
GSA: Genome Sequence Archive
GSEA: Gene set enrichment analysis
LH: Luteinizing hormone
MSigDB: Molecular Signatures Database
NCT: Null cell tumor
NK: Nature killer
ORA: Over representation analysis
PCA: Principal component analysis
PCs: Principal components
PitNETs: Pituitary neuroendocrine tumors
PRL: Prolactin
scRNA-seq: Single-cell RNA sequencing
TAMs: Tumor-associated macrophages
TCGA: The Cancer Genome Atlas
TF: Transcription factor
TME: Tumor microenvironment
TSA: Tyramide signal amplification
TSH: Thyroid-stimulating hormone
UMAP: Uniform Manifold Approximation and Projection
