# Supplementary material for: Single-cell transcriptomic analysis reveals tumor cell heterogeneity and immune microenvironment features of pituitary neuroendocrine tumors
Source: Genome Med. 2024 Jan 2;16:2. doi: 10.1186/s13073-023-01267-3 (PMC10759356; doi:10.1186/s13073-023-01267-3)
Supplement: Supplementary file 1 — Additional file 1: Figure S1-S12. All supplementary figures. [file 13073_2023_1267_MOESM1_ESM.docx]

# Supplementary figures


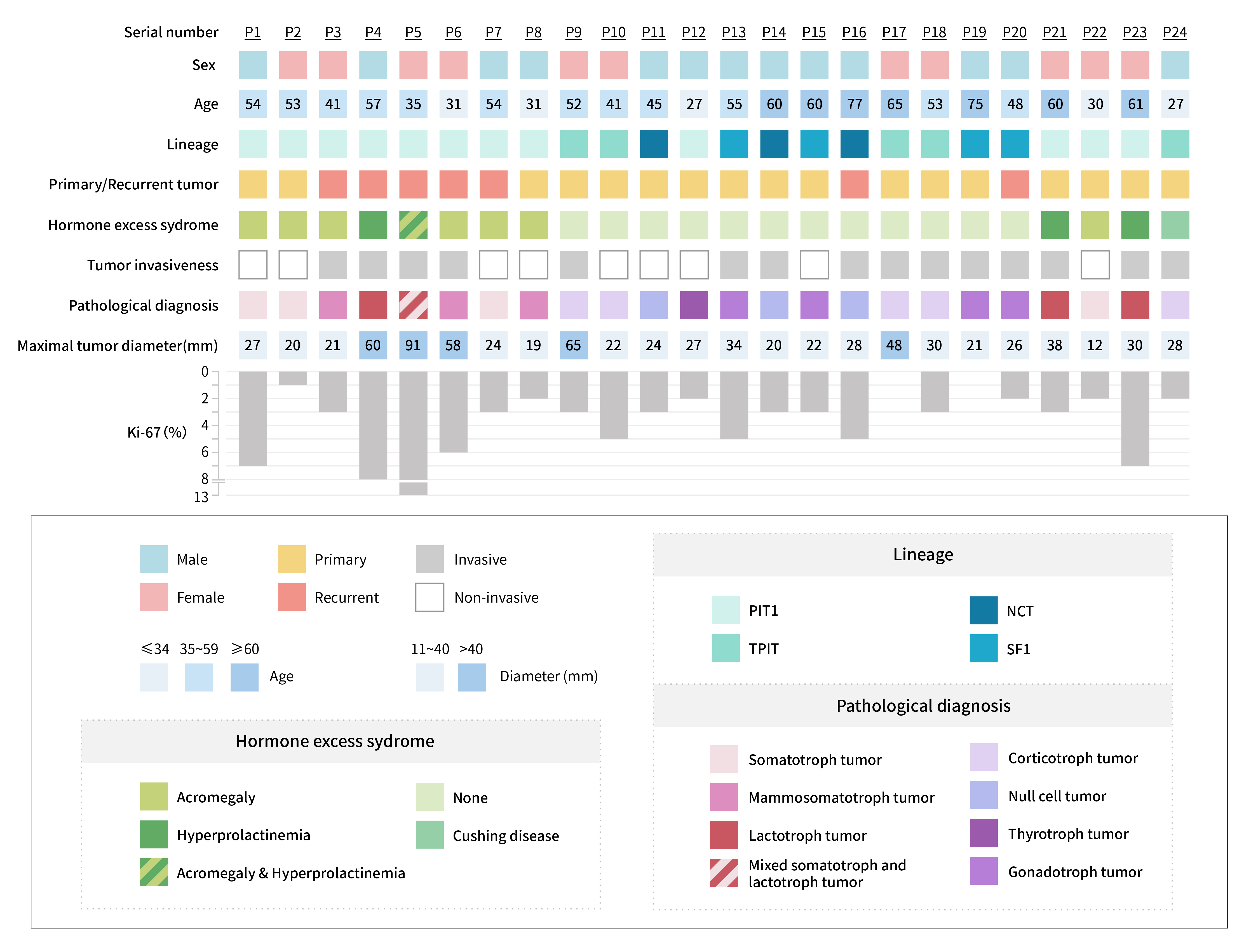


**Figure S1 Clinical details of the 24 pituitary neuroendocrine tumor (PitNET) patients.** The lineage of PitNET is labelled according to the 2022 WHO classification criteria of pituitary tumors. Specifically,

- PIT1-lineage PitNET: including somatotroph tumor, mammosomatotroph tumor, lactotroph tumor and thyrotroph tumor. (PIT1)
- TPIT-lineage PitNET: including corticotroph tumor. (TPIT)
- SF1-lineage PitNET: including gonadotroph tumor. (SF1)
- Null cell tumor (NCT).


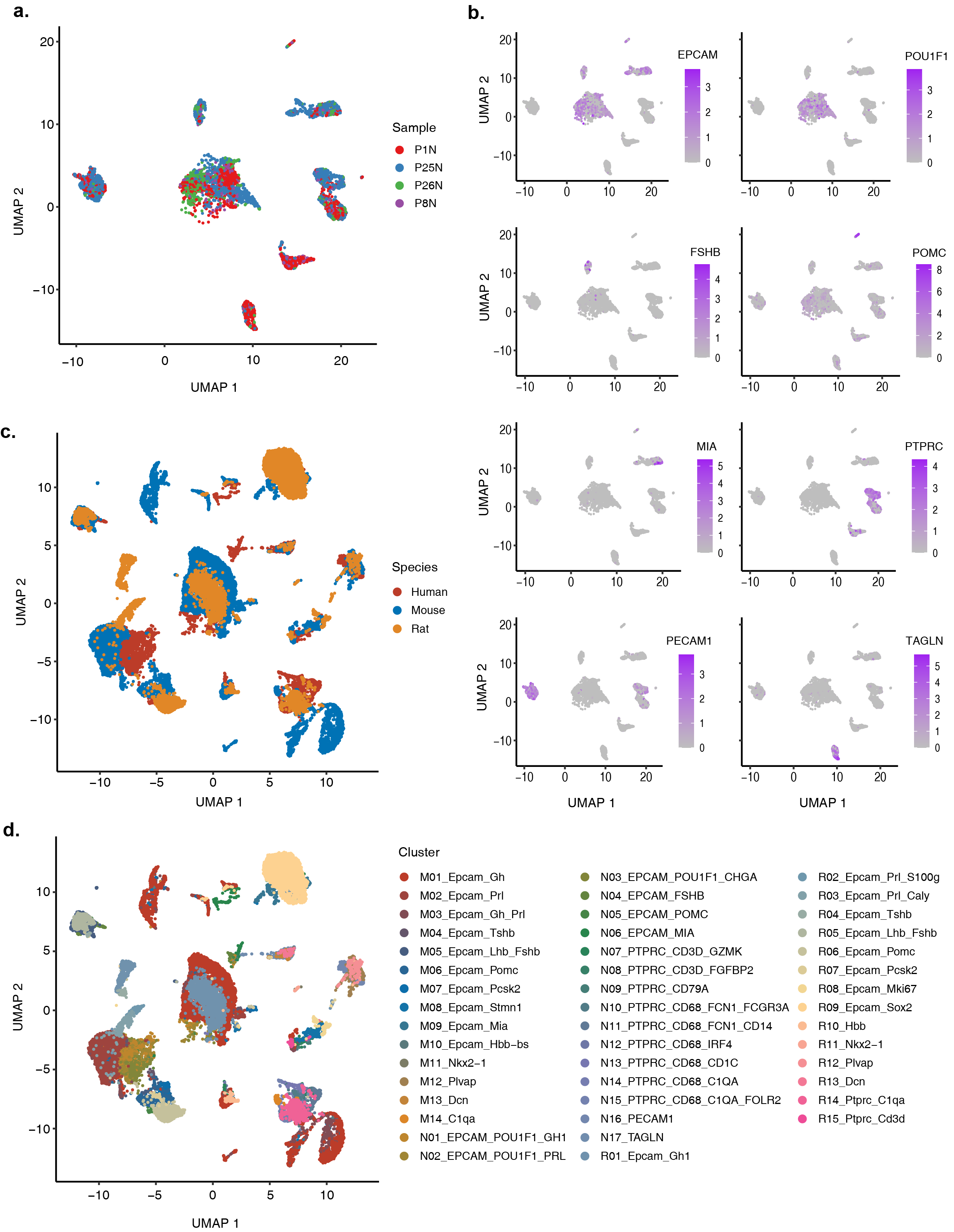


**Figure S2 Single-cell landscape of normal human pituitary and comparison with mouse and rat pituitary. a** UMAP plot showing the batch label of normal pituitary cells. **b** Expression level of specific marker genes on UMAP plot. **c** UMAP plot showing the batch label of the integrated pituitary atlas. **d** UMAP plot showing the annotated cell type in the integrated pituitary atlas.


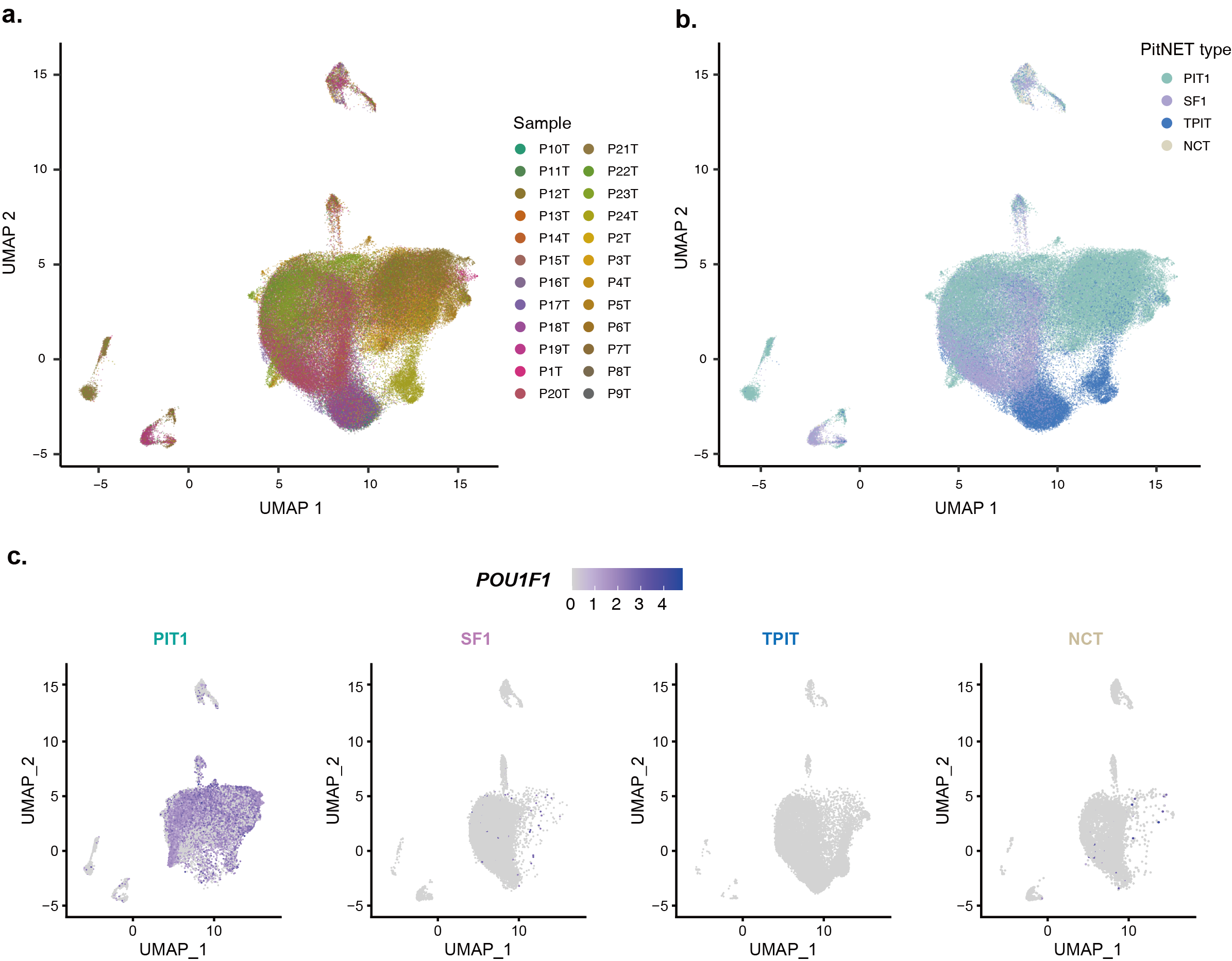


**Figure S3 Characteristics of** PitNET **samples. a** UMAP plot showing the batch label of PitNET cells. **b** UMAP plot showing the PitNET types of pituitary tumor cells. **c** UMAP plot showing the expression of *POU1F1* across four types of PitNET samples.

**
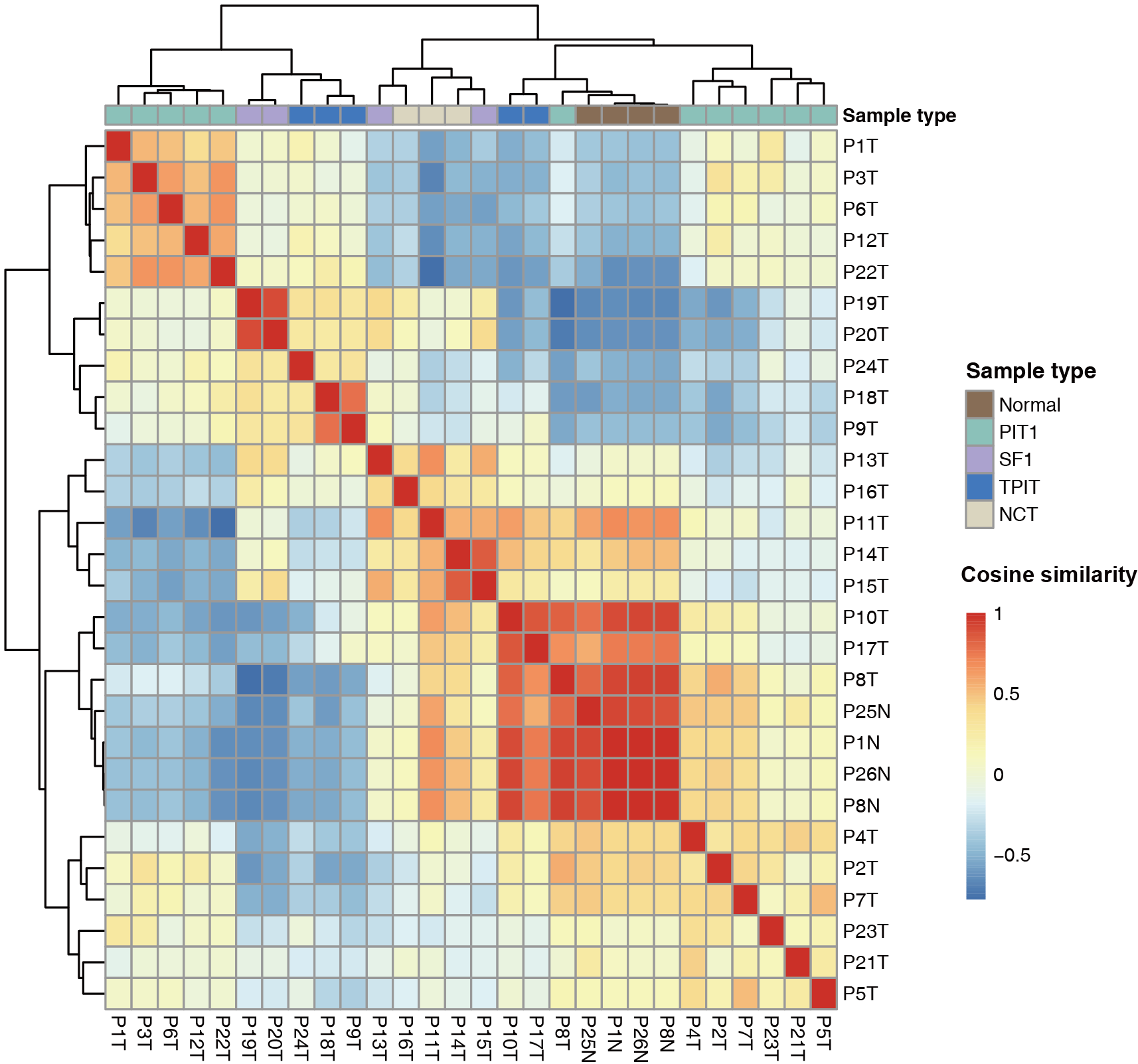
**

**Figure S4 Pairwise cosine similarities of all pituitary samples.** The rows and columns are arranged based on hierarchical clustering.


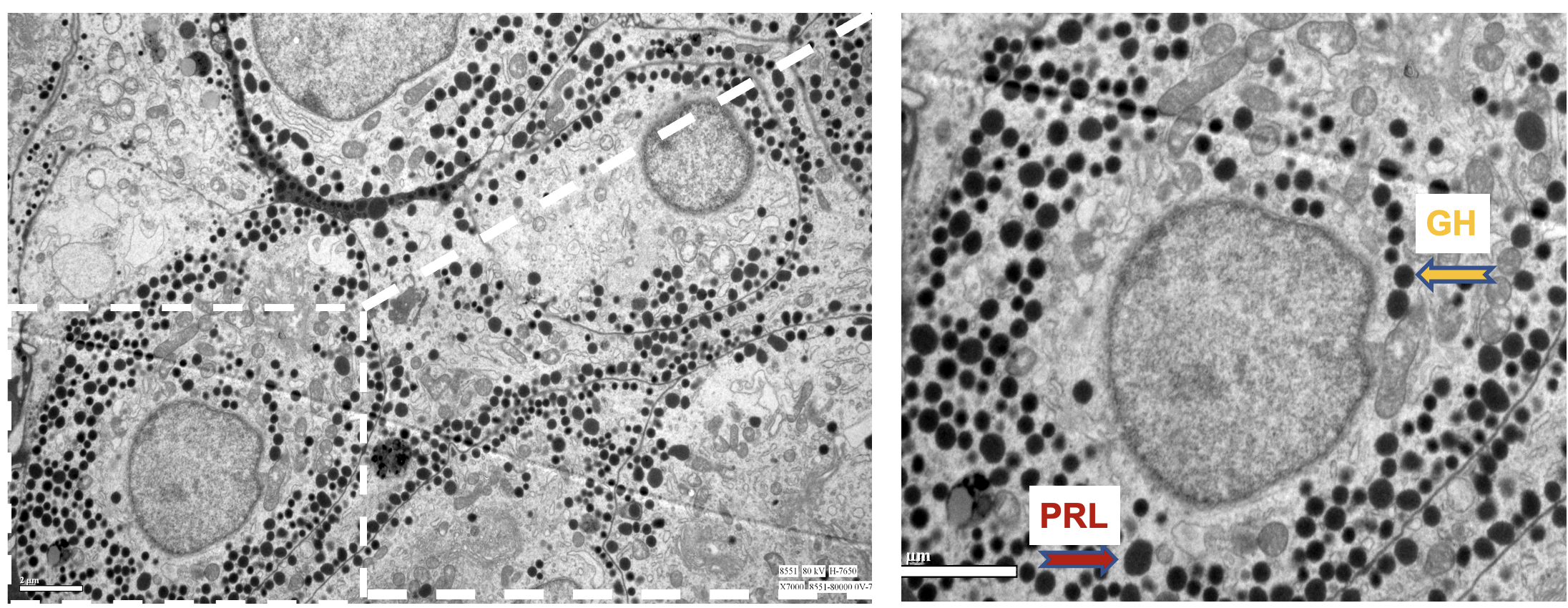


**Figure S5 The electron micrograph of typical lactotrophs and somatotrophs secretory granules of P3T PitNET.** Prolactin (PRL) cells: The granules typically have diameters ranging from 350 to 550 nm. They are irregular in shape. Growth Hormone (GH) cells: The granules in these cells have a diameter ranging between 250 to 400 nm and are round in shape. Scale bar: 2 µm


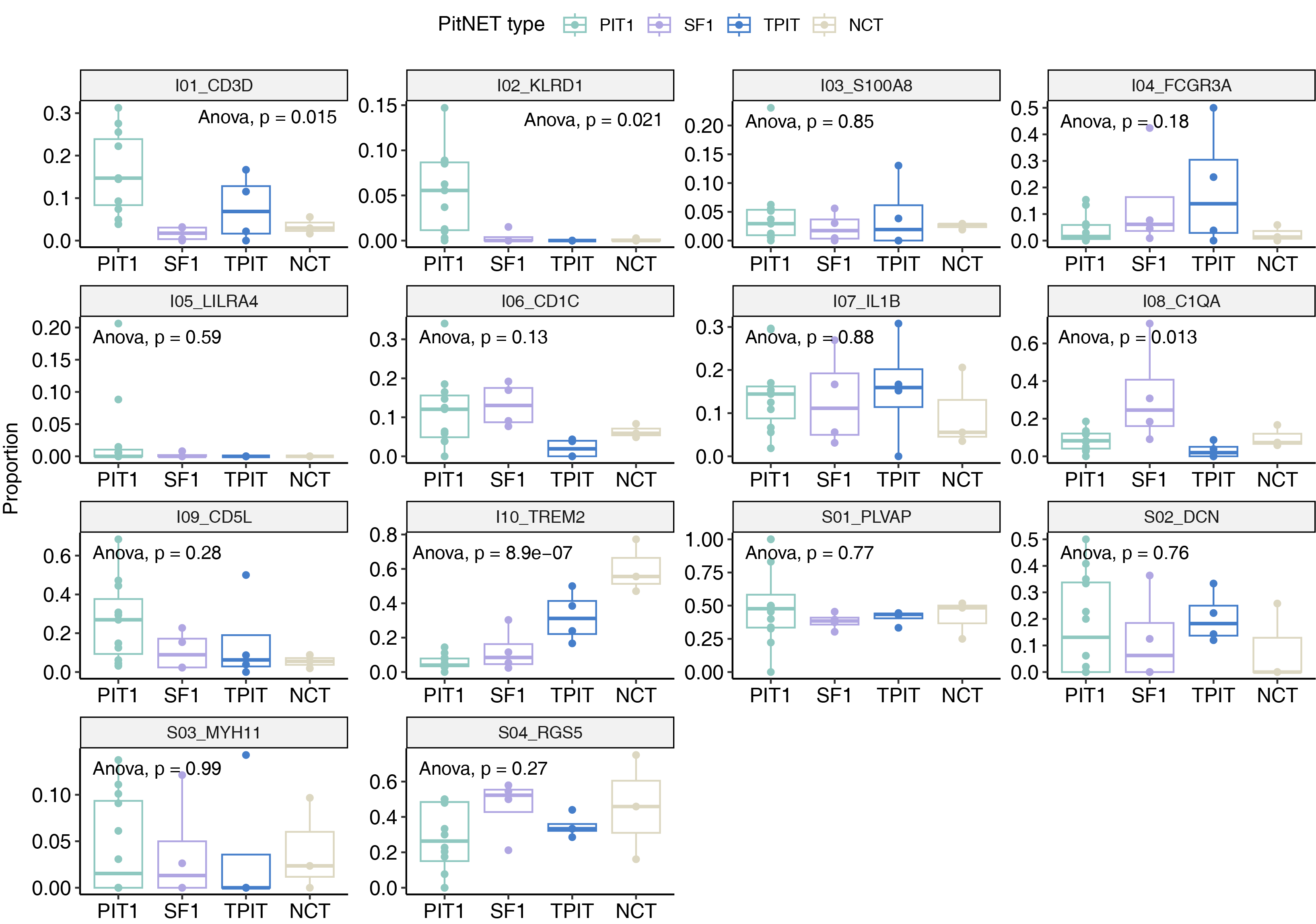


**Figure S6 Box plot showing the proportion of immune and stromal subpopulations across four types of PitNETs.**

**
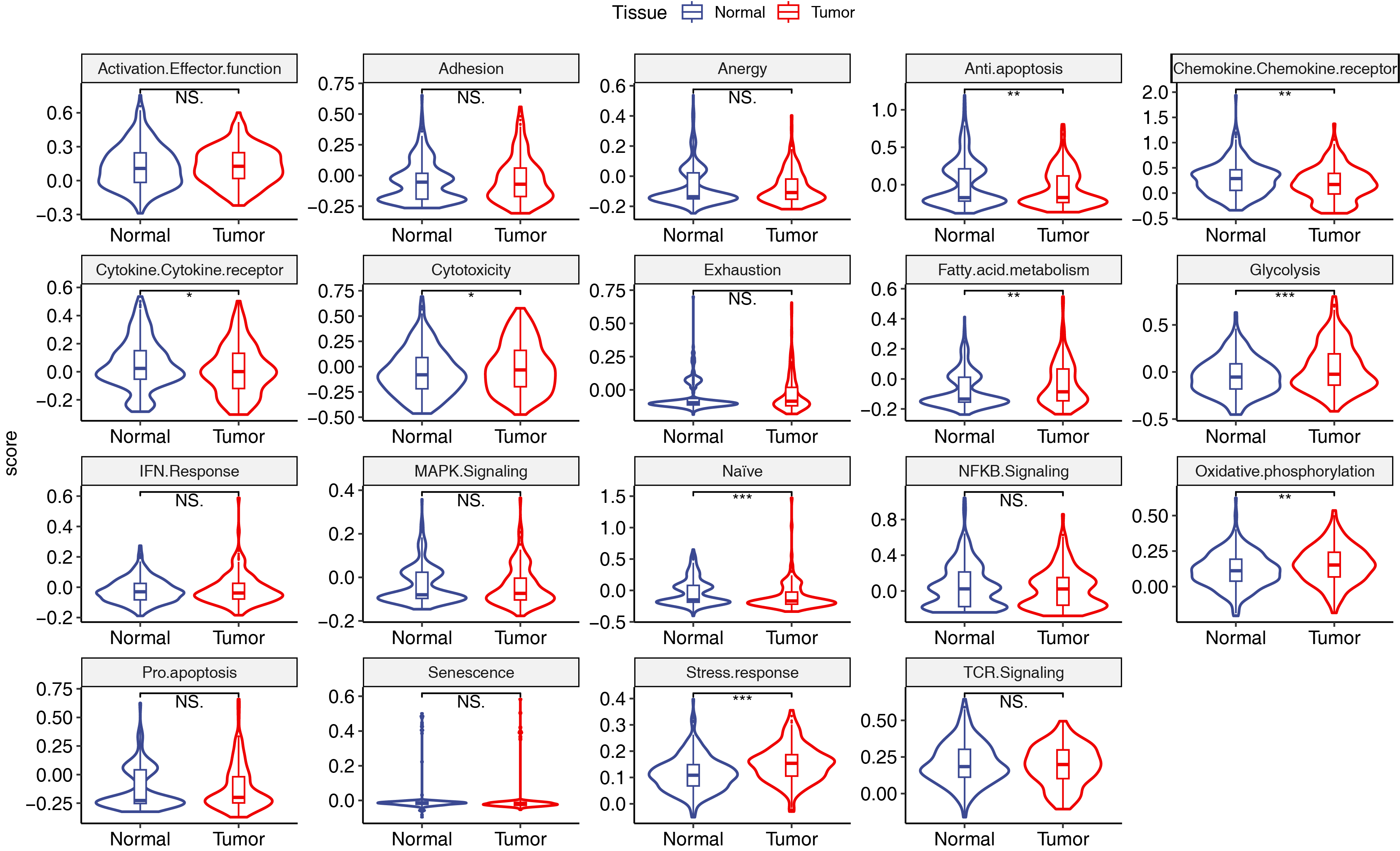
**

**Figure S7 Box plot showing the functional signature scores of T cells between normal pituitary and PitNETs.** * p < 0.05, ** p < 0.01, *** p < 0.001. NS., not significant. Student’s t-test.


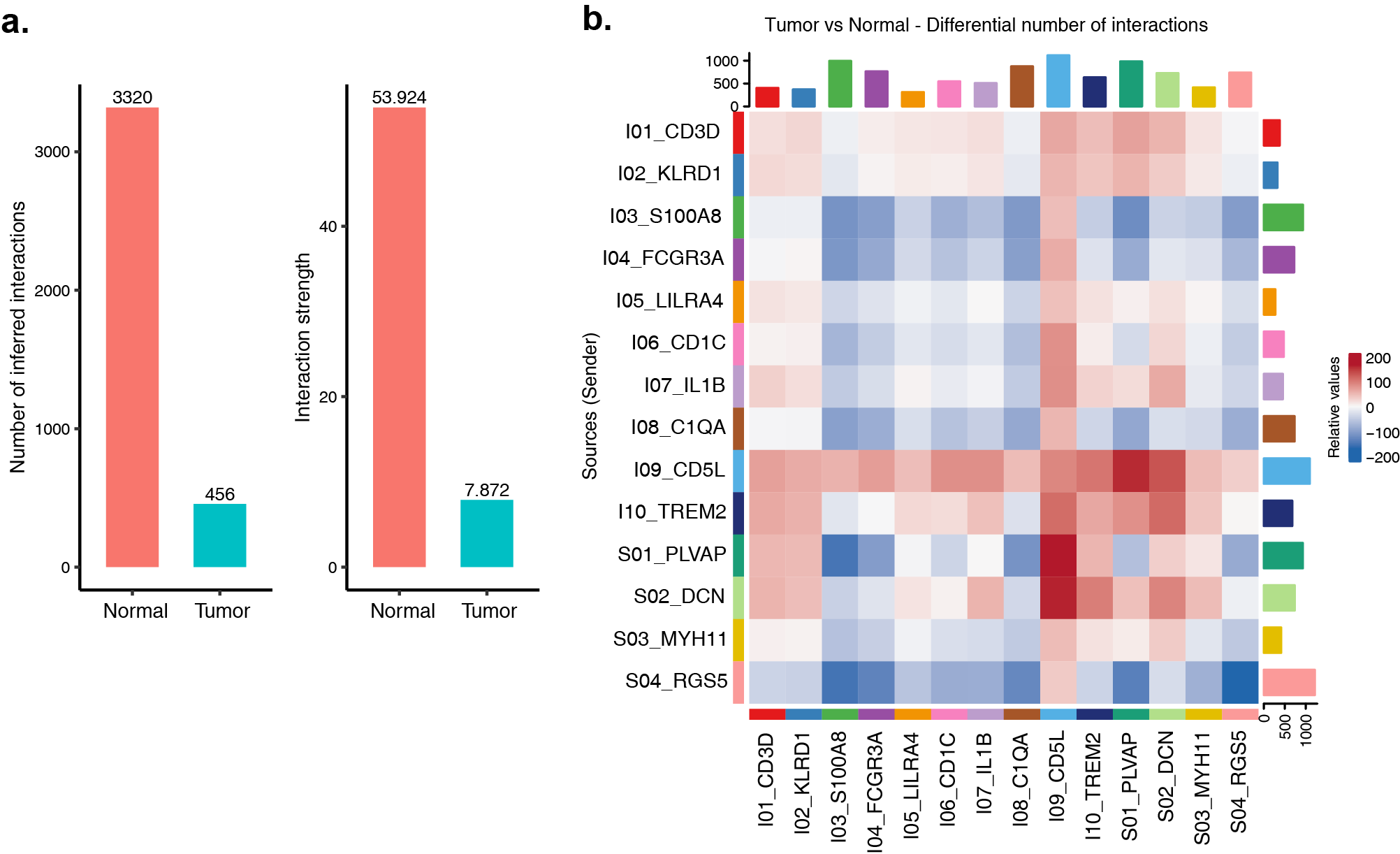


**Figure S8 Difference of cellular interaction between normal pituitary and PitNETs.** **a** Bar plot showing the number of inferred interaction and strength. **b** Heatmap showing the differential number of interactions between tumor and normal pituitary. Top and right bars show the cumulative signal strength in each cellular population.


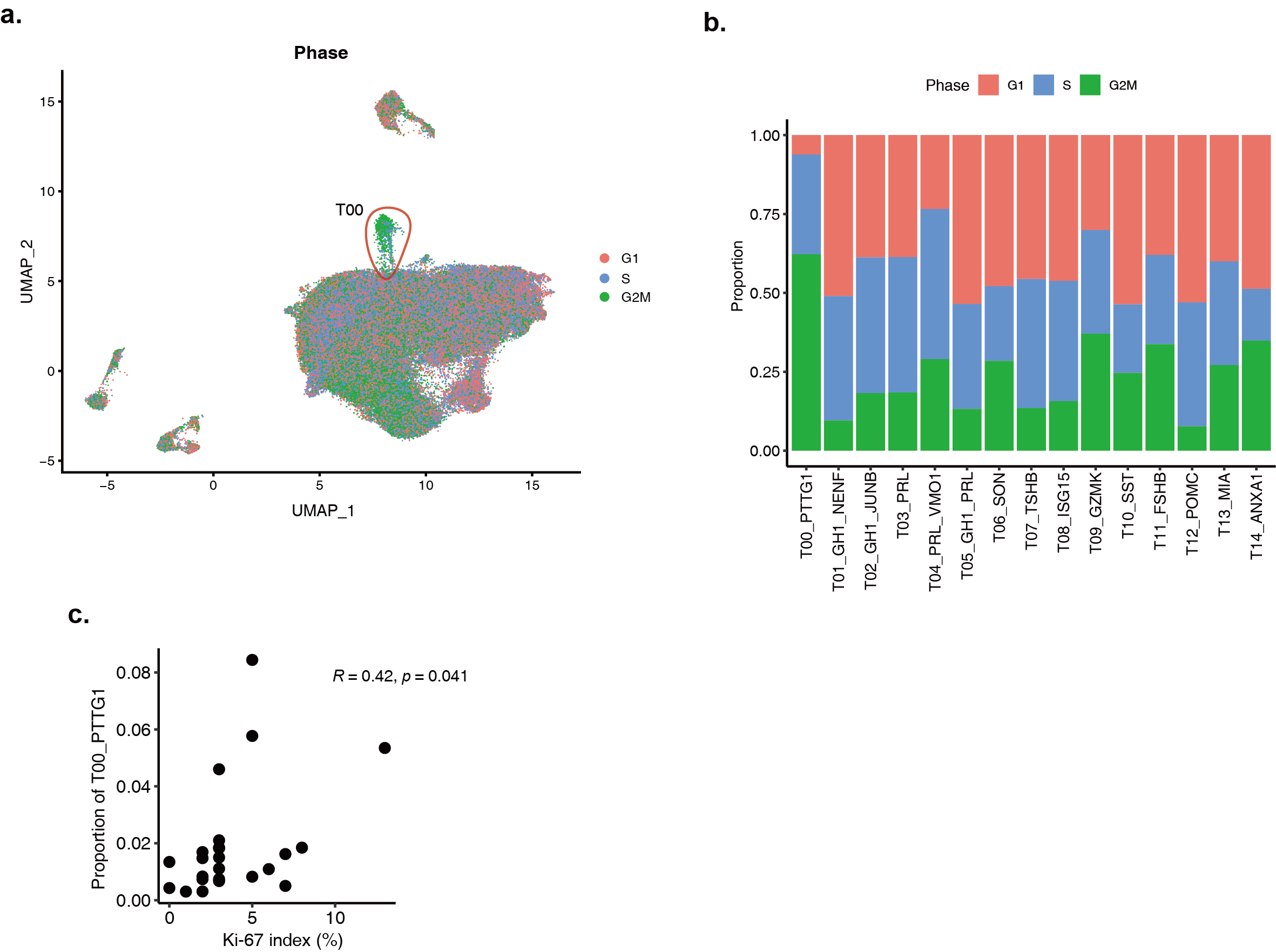


**Figure S9 characterization of the aggressive cellular subpopulation.** **a** UMAP plot labeled by inferred cell cycle. **b** Histogram showing the proportion of different cell cycle in all clusters. **c** Scatter plot showing the relationship between the proportion of Cluster T00 and the Ki-67 index examined in clinical. Pearson correlation.


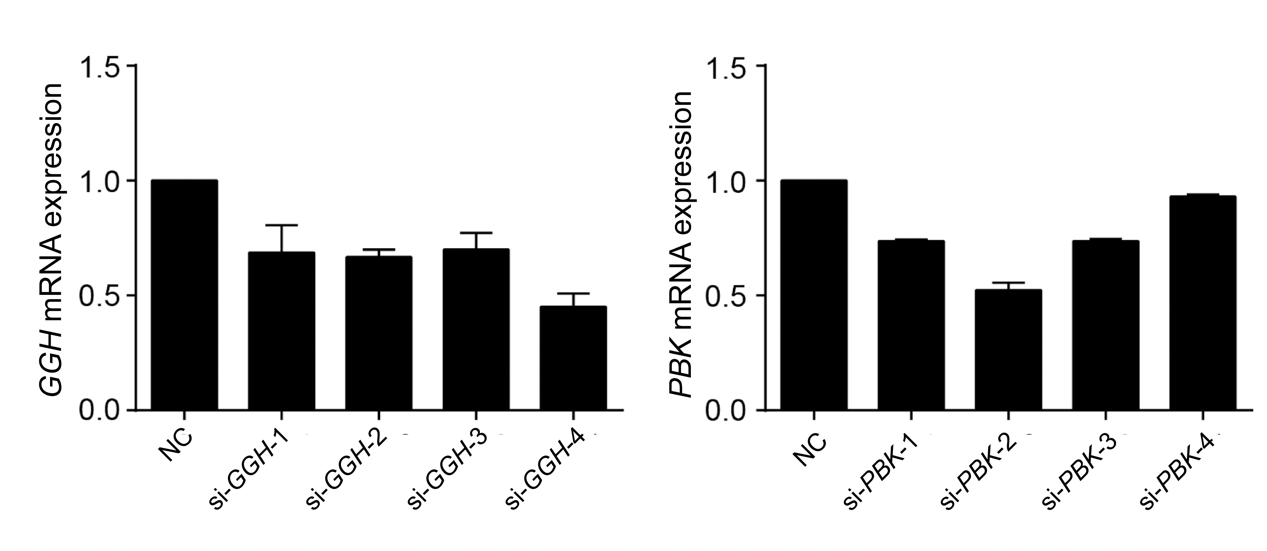


**Figure S10 Expression levels of *GGH* and *PBK* after different siRNAs’ transfections in the experiment of cell proliferation.** (normalized by NC)


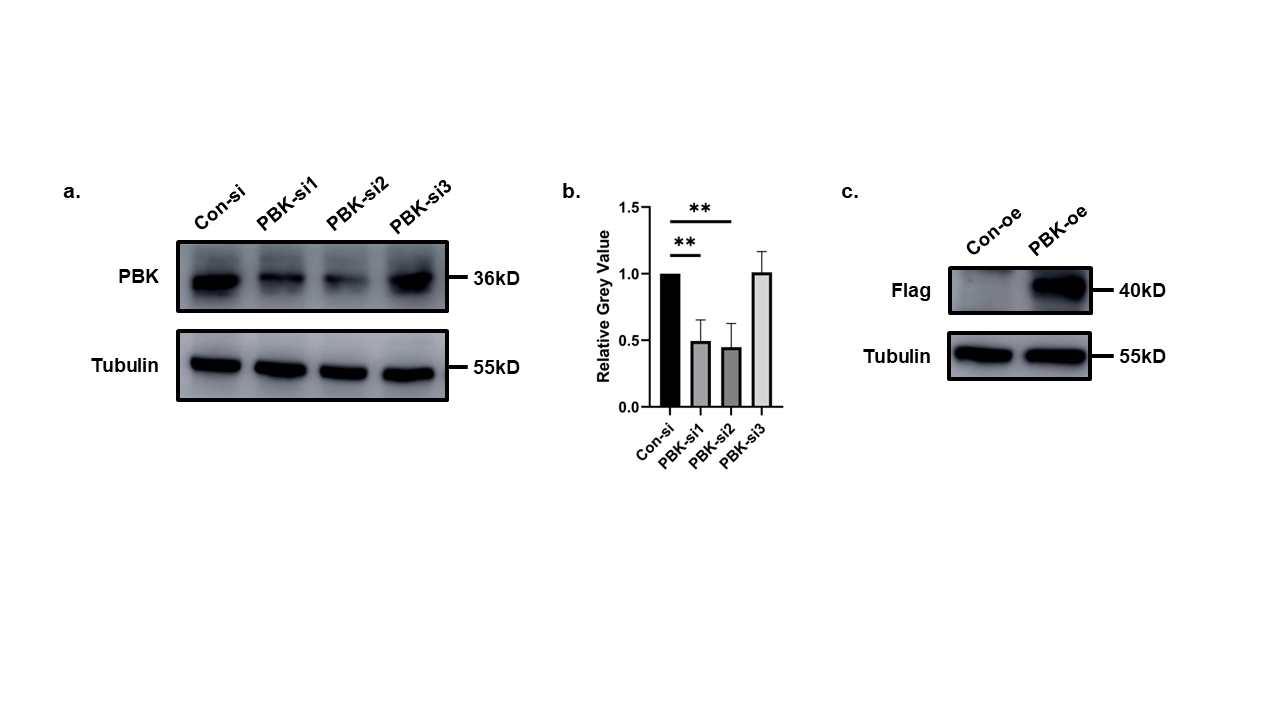


**Figure S11 Validation of transwell experiment for cell migration in GH3 cells. a** Transfection efficiency of *PBK* gene was assessed by western blot, versus control. **b** Quantification of the protein expression levels of *PBK* by Western blot. Band intensities were quantified and standardized to tubulin. **c** Transfection efficiency of *PBK* gene was assessed by western blot. ** p < 0.01, *** p < 0.001. One-way ANOVA.

**
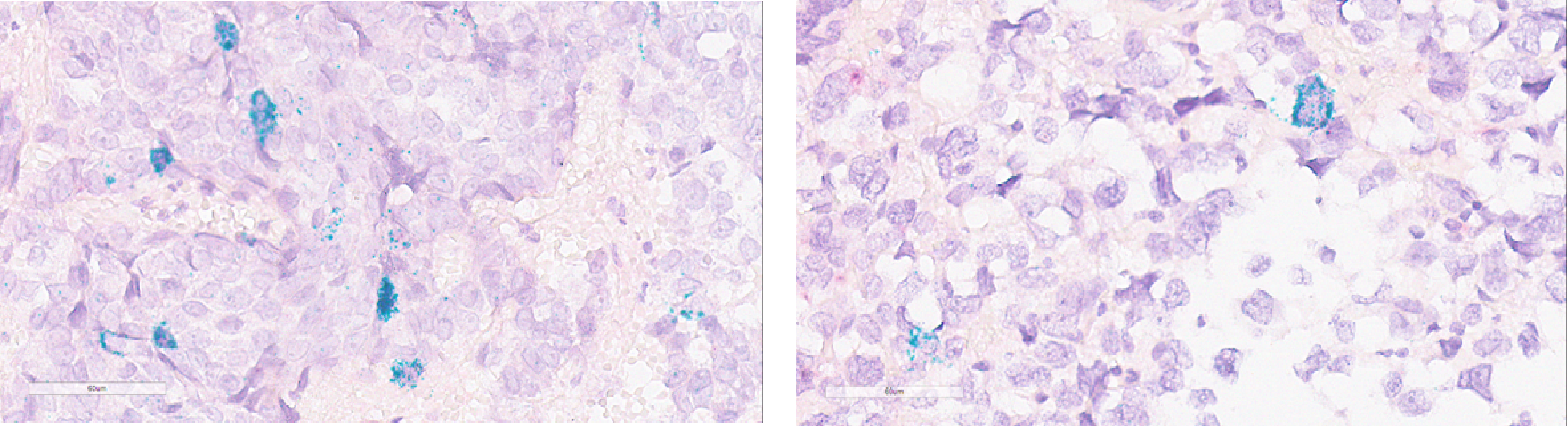
**

**Figure S12 RNAscope staining of PBK in PitNET tissues.** Representative images illustrating the RNAscope staining of *PBK* in PitNET samples. Green granules indicate pituitary tumor cells that were positively stained for *PBK* RNA using RNAscope. Scale bars, 60 µm.
